# Loss of early B cell protein λ5 decreases bone mass and accelerates skeletal aging

**DOI:** 10.3389/fimmu.2022.906649

**Published:** 2022-09-14

**Authors:** Mohamed Khass, Harunur Rashid, Peter D. Burrows, Amjad Javed, Harry W. Schroeder

**Affiliations:** ^1^ Department of Medicine, University of Alabama at Birmingham, Birmingham, AL, United States; ^2^ Department of Oral and Maxillofacial Surgery, University of Alabama at Birmingham, Birmingham, AL, United States; ^3^ Department of Microbiology, University of Alabama at Birmingham, Birmingham, AL, United States

**Keywords:** λ5, B cells, bone mass, skeletal aging, Trabecular bone recovery

## Abstract

The early B cell protein λ5 is an essential component of the surrogate light chain and the preB cell receptor (preBCR), which is critical for optimal B cell development. To investigate the effect of λ5 and/or B cells on bone acquisition over time, we developed a panel of *J_H_^-/-^*, λ5^-/-^, *J_H_^-/-^* λ5^-/-^, and wild-type (WT) BALB/c mice and then studied postnatal bone development and aging in these mice at one, six, twelve, and twenty-two months of age. The trabecular bone volume over total volume (BV/TV) in *J_H_^-/-^* mice was similar to WT mice at all ages. In contrast, at six months of age and thereafter, λ5^-/-^ and *J_H_^-/-^* λ5^-/-^ mice demonstrated a severe decrease in trabecular bone mass. Surprisingly, bone mass in six-month-old λ5^-/-^ and *J_H_^-/-^* λ5^-/-^ mice was similar to or even lower than in aged (twenty-two-months) WT mice, suggesting accelerated skeletal aging. The postnatal development and the acquisition of cortical bone mass in *J_H_^-/-^* λ5^-/-^ mice were generally comparable to WT. However, *J_H_^-/-^* λ5^-/-^ mice showed a significant decrease in cortical BV/TV at six- and twelve months of age. To examine the contribution of λ5 and B cells to postnatal bone synthesis, we separately transplanted whole bone marrow cells from *J_H_^-/-^* λ5^-/-^ and WT mice into irradiated *J_H_^-/-^* λ5^-/-^ and WT recipients. WT recipients of *J_H_^-/-^* λ5^-/-^ marrow cells failed to show acquisition of trabecular bone mass, whereas transplanting WT marrow cells into *J_H_^-/-^* λ5^-/-^ recipients led to the recovery of trabecular bone mass. Transfer of WT marrow cells into *J_H_^-/-^* λ5^-/-^ mice promoted synthesis of new cortical and trabecular bone. Our findings indicate that λ5 plays a major role in preserving bone mass during postnatal development and skeletal aging which is distinct from its role in B cell development. The absence of both λ5 and B cells in *J_H_^-/-^* λ5^-/-^ mice leads to delayed acquisition of cortical bone during postnatal development. Dissecting the mechanism(s) by which λ5 regulates bone homeostasis may provide new avenues for the treatment of age-related loss of bone mass and osteoporosis.

## Introduction

The strength and mass of cortical and trabecular bone decrease with aging, especially among women (1). For example, more than half of all women aged 50 and over had evidence of low bone mass and one in five women suffered from overt osteoporosis (2). Loss of bone mass and structural integrity are key risk factors for fractures, which can lead to pain, disability and even death (3). A major cause of the loss of bone structural integrity is a decline in bone formation due to decreased osteoblast numbers and activity, coupled with an increase in bone resorption. Our knowledge of B cell products that influence gross changes in bone mass with age is incomplete, which complicates the development of approaches to block and preferably reverse the effects of aging on bone.

Important regulatory interactions have been shown to occur between the cells of the immune and skeletal systems (4). B cell progenitors arising from hematopoietic stem cells develop in supportive niches located on endosteal bone surfaces that surround the bone marrow. Mature B cells produce cytokines and chemokines that are involved in bone homeostasis, including but not limited to tumor necrosis factor alpha (TNFα), interferon gamma (IFNγ), osteoprotegerin (OPG), and RANK ligand (RANKL) (5).

Early B cell differentiation focuses on the generation and selection of immunoglobulin mu heavy (H) chains (μHC). VDJ recombination at the heavy chain locus begins in progenitor (pro) B cells. These cells express λ5 and VpreB, which associate to create a noncovalent, heterodimeric surrogate light chain (SLC) that can take the place of a conventional light (L) chain (LC). Expression of μHC protein after successful VDJ recombination defines development to the early preB cell stage. Selective exon splicing can create a μHC with either a transmembrane anchor or a secretory terminus. Progression from the early to late preB cell stage is promoted by successful association of SLC with μHC to form a preB cell receptor (preBCR), which also contains the transmembrane chaperone/signaling molecules Igα and Igβ (CD79a/b). CD19, a pan-B lineage transmembrane glycoprotein, can augment signaling through the preBCR. Activation of the preBCR is accompanied by termination of SLC production and HC gene rearrangement, proliferation and initiation of LC gene rearrangement which, when successful, permits expression of a complete, membrane-bound IgM B cell receptor (BCR) on the surface of the immature B cell.

In previous studies of the role of the preBCR in restricting the diversity of the immunoglobulin HC repertoire, we observed that the bones of six-month-old mice deficient in λ5 (λ5^-/-^) were more fragile than WT (6). Working with a C57BL/6 panel of six-month-old λ5^-/-^, IgM transmembrane-deficient (µMT^-/-^) and CD19-deficient (CD19^-/-^) mutants, we observed loss of trabecular and cortical bone in the mutants when compared to WT controls (6). λ5^-/-^ mice cannot form a preBCR and there is a block in B cell development starting from early preB cells (7). However, this mutation is ‘leaky’ and a small number of late preB cells can bypass the B cell developmental block even though they lack λ5 expression, presumably by precocious light chain gene expression before heavy chain gene rearrangement (8). In the periphery, a full panoply of mature B cell subsets in (λ)5 deficient mice is observed and has been noted to normalize in number with aging. µMT^-/-^ mice can form secretory μHC that associates with SLC but they cannot form a membrane-bound preBCR or BCR, leading to a complete loss of mature B cells and their progeny (9). Coupled with a trend for decreased bone in CD19^-/-^ mice, in which preBCR and BCR signal strength after activation is diminished, these findings suggested that λ5 might be playing a critical role in bone formation, potentially through a mechanism involving the preBCR. Support for this hypothesis comes from the finding that progenitor B cell numbers and λ5 expression decrease with age in both mice and humans (10–23).

To test whether λ5 and μHC might either be a part of the same preBCR pathway or might play separate but complementary roles in bone homeostasis with age, we prepared a panel of *J_H_^-/-^*, and λ5^-/-^ single mutants, and *J_H_^-/-^* λ5^-/-^ double mutants on a BALB/c background. In our previous studies of C57BL/6 mice, we used µMT^-/-^ mutants as a model for complete B cell deficiency; however, in BALB/c mice this mutation is ‘leaky’ (24). Class switching permits formation of an IgG-containing BCR and mature B cell subsets and their progeny, a phenotype that would complicate interpretation of our results. *J_H_^-/-^* B lineage cells lack all four J_H_ gene segments, cannot produce immunoglobulin HC proteins, and thus cannot associate with SLC even in their secretory form (32). Therefore, we elected to use *J_H_^-/-^* BALB/c mice, wherein, by definition, B cell development terminates at the proB cell stage (**Supplementary Figure S1**). These mice cannot produce mature B cells that express tumor necrosis factor alpha (TNFα), interferon gamma (IFNγ), osteoprotegerin (OPG), or RANK ligand (RANKL) (5). Double mutant *J_H_^-/-^* λ5^-/-^ mice produce proB cells that both lack λ5 expression and are devoid of all B cell subsets past this proB cell stage, including preB cells, immature B cells, mature B cells, and plasma cells.

Both BALB/c and C57BL/6 strains have been frequently used to model and dissect the effect of aging on bone (1, 4, 26, 27). In both strains, bone structure develops rapidly during the first two months of life, reaching peak bone mineral density and cortical size by six-months of age (1). Mice 18-24 months of age are considered ‘old’. Bone mass is assessed as the ratio of bone volume over total volume (BV/TV), which is an indicator of the actual bone volume relative to non-bone elements and cells (28). In WT mice, trabecular BV/TV declines steadily after 1.5 to 2 months of age (25), with a 50-60% loss in old mice when compared to the young (29).

We evaluated trabecular and cortical bone mass at one, six, twelve, and twenty-two-months in the above panel of mice and found that λ5 and B cell lineages beyond the proB cell stage, play different roles in the acquisition and loss of bone as a function of age. Strikingly, trabecular bone loss was ameliorated, indeed underwent reversal, when WT bone marrow was transplanted into *J_H_^-/-^* λ5^-/-^ mice. These findings could lead to new approaches to prevent or even reverse loss of bone mass during aging.

## Materials and methods

### Mouse models

Loss of bone mass with age occurs more frequently and more severely in women, and thus all of our experiments were performed with female BALB/c mice. The generation of *J_H_^-/-^* and λ5^-/-^ mice on a C57BL/6 background has been previously reported (7, 32). These two strains were backcrossed for 22 generations onto a BALB/c background and then intercrossed to obtain homozygous *J_H_^-/-^* λ5^-/-^ mice. Genotypes were verified by PCR using DNA from tail biopsies with direct PCR lysis reagent (Viagen Biotech; Los Angeles, CA). The targeted alleles were confirmed by using gene-specific primer pairs and by evaluation of B cell populations by flow cytometry. Deletion of the *Igll1* gene (λ5) was confirmed by genomic PCR using the forward primer 5’GGAGATCTACACT GCAAGTGAGGCT 3’ and reverse primer 5’ACACTGGCCTTGCAATTGATCGAG 3’ and the absence of λ5 expression was verified by flow cytometry (**Supplementary Figure S2**).

All mice were housed under a twelve-hour light:dark cycle with *ad libitum* access to food and water. To evaluate postnatal bone development and homeostasis, limbs were harvested at one-, six-, twelve-, and twenty-two-months of age. Femurs were analyzed using µCT and histomorphometry (6). For the bone marrow transplantation experiments, three-month old mice were irradiated. Whole bone marrow cells were harvested from two-month old mice and used for transplantation into irradiated recipient mice. All experiments were performed after approval from the Institutional Animal Care and Use Committee of the University of Alabama at Birmingham and conformed to relevant federal and state guidelines and regulations.

### µCT analysis

Three-dimensional bone structure and mineral density were assessed by micro-computed tomography (µCT). Femurs were dissected and scanned using the µCT40, e beam system (Scanco Medical AG, Brüttisellen, Switzerland).

### Histological processing and histomorphometry analysis

For histomorphometric analysis, 7 µm lateral sections of undecalcified femur were processed and stained with Masson’s trichrome or TRAP. Analyses on lateral sections midway through the femur were performed using the Bioquant Osteo semi-automated system for skeletal phenotyping. Nomenclature, symbols, and units used are those recommended by the Nomenclature Committee of the American Society for Bone and Mineral Research (30). All histomorphometric analyses were performed on three independent sections and assessed by blinded examiners.

To perform histomorphometric analysis of osteoblast and osteoclast parameters, femur sections were stained with Masson’s trichrome and imaged using a Nikon microscope (Nikon Eclipse 80i color camera). For quantification of trabecular bone, a 250 micron region beneath the growth plate and a depth of 1 millimeter off of the peak of the growth plate region was selected as a region of interest. All of the trabecular bone in the region was traced and measured for volume and bone surface. Bioquant Osteo v18.2.60 software (BIOQUANT Image Analysis Corporation, Nashville, TN) was used to take the measurements for the bone parameters. Enumeration was performed by three blinded evaluators and the data were averaged to minimize personal bias.

### Irradiation, adoptive transfer, and dynamic bone synthesis analysis

Three-month old mice received two doses of 600 rads at 3-4 hours intervals (fractionated dose: 600 + 600 with 3-4 h interval) from an X-ray source as described previously (31). This dose resulted in complete myeloablation for both the WT mice and the immunocompromised *J_H_^-/-^* λ5^-/-^ mice that lack B cells. Irradiated mice were then intravenously injected with 5 x 10^6^ total bone marrow cells harvested from two-month old mice. Mice were maintained for six months after the adoptive transfer to allow for tissue reconstitution and proper engraftment. One week and then two days before euthanasia, mice received intraperitoneal injections of 20 mg of calcein (Sigma-Aldrich) per kg of body weight in a 2% sodium bicarbonate solution. Limbs were collected and processed two days after the second calcein injection. Histologic, histomorphometry and μCT analysis were performed on the same bone sets of adoptively transferred mice. To screen for successful transfer and engraftment after transplantation of WT BM into *J_H_^-/-^* λ5^-/-^ mice, we collected peripheral blood and confirmed the presence of the λ5 gene (*Igll1*) using genomic PCR.To calculate the mineral apposition rate (MAR) and the bone formation rate (BFR), calcein label was measured from unstained femur sections under fluorescent light. Parameters of dynamic bone formation were assessed using Bioquant Osteo v18.2.60 software as published previously (6).

### Statistical analysis

Statistical power was calculated assuming Analysis of Variance (ANOVA) comparing outcomes across 4 groups with the sample size per group varying by experiment, some experiments having 3 animals per group, some experiments having 4 per group, and other experiments having 5 animals per group. Assuming a Type I error rate of 0.05 and comparison of 4 groups, 3 animals per group (total n = 12) would provide 80% power to detect an R^2^ of 60% (i.e. 60% of the variance is due to variation in group means). Under the same assumptions, 4 animals per group (total n = 16) would provide 80% power to detect an R^2^ of 48.5%; 5 animals per group (n = 20) would have 80% power to detect an R^2^ of 41.3%. All calculations were done using PASS 14.0. (PASS 14 Power Analysis and Sample Size Software (2015). NCSS, LLC. Kaysville, Utah, USA, ncss.com/software/pass.)

Differences in bone parameters among various genotypes were assessed by one-way ANOVA. Analysis was performed with JMP Statistical Discovery software (SAS Institute, Inc., Cary, NC). Mean values were calculated along with the standard error of the mean.

## Results

To test the influence of λ5 on bone homeostasis and aging, we created a panel of BALB/c mice with mutant-specific blocks in B cell development at key developmental checkpoints (**Supplementary Figure S1**). λ5^-/-^ mice lack λ5 expression and have an incomplete block in B cell development at the late preB stage that results in diminished passage of immature and mature B cells through the preBCR checkpoint. Previous studies of λ5^-/-^ C57BL/6 mice showed that by ~six-months of age, these mice have a mature B cell pool similar in number to WT (6, 7). To study bone mass in the absence of B cells but in the presence of λ5, we used mice lacking all four J_H_ gene segments (*J_H_^-/-^*). Developing B cells in these mice cannot undergo VDJ_H_ rearrangement and thus cannot form a μHC. They are devoid of preB, immature B, mature B, and plasma cells (32). This phenotype is hereafter referred to as post proB deficient. However, B cells at the progenitor stage (proB) continue to produce λ5 as its expression is initiated prior to D_H_→J_H_ recombination. To study bone in the absence of both λ5 and B lineage cells post proB, we crossed *J_H_^-/-^* and λ5^-/-^ mice to generate *J_H_^-/-^* ${\lambda 5}^{-/-}$ double knockout mutants.

### Loss of the λ5 gene and early/immature/mature B cells leads to a significant decrease in adult bone mass

**Figure 1:**
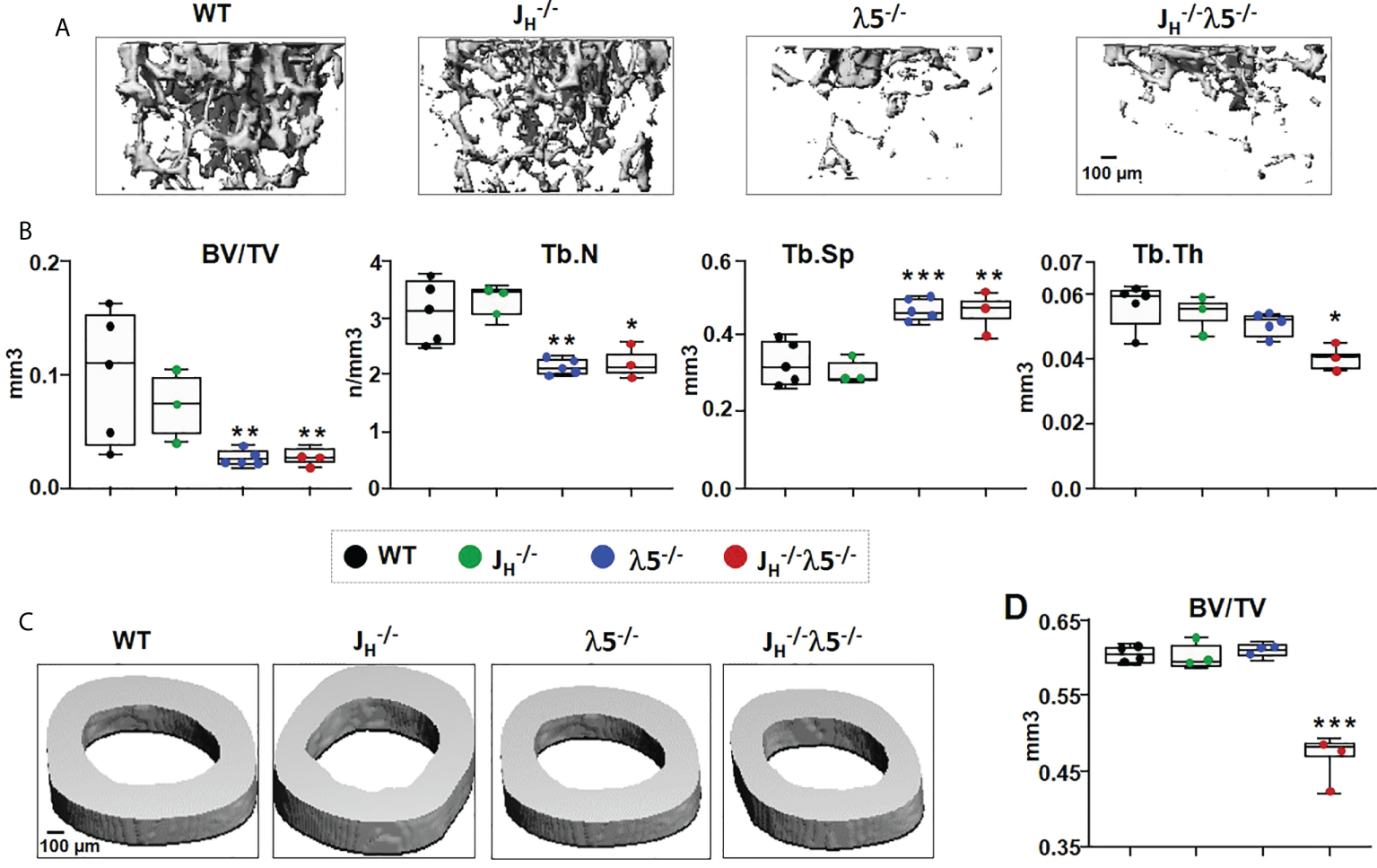
Deficiency of λ5 and/or B cells decreases adult bone mass. **(A)** Representative µCT images of trabecular bone beneath the femoral growth plate and **(C)** cortical bone at the mid-diaphysis from the six-month old WT, *J_H_^-/-^* λ5^-/-^ and *J_H_^-/-^* λ5^-/-^ BALB/c female mice. **(B)** Quantification of the data from each genotype used to determine the bone parameters; ratio of trabecular bone volume to tissue volume (BV/TV), trabecular numbers (Tb.N), trabecular spacing (Tb.Sp), and trabecular thickness (Tb.Th). **(D)** The ratio of cortical bone volume to tissue volume (BV/TV) for each genotype. Data presented are pooled from n=3-5 mice per group. Statistical significance relative to WT mice was calculated by ANOVA. *p<0.05, **p<0.01, ***p<0.001. Scale bar: 100µm.

To examine the influence of λ5 and/or developing post proB cells on the adult bone mass, we studied six-month old WT, *J_H_^-/-^*, λ5^-/-^, and *J_H_^-/-^* ${\lambda 5}^{-/-}$ female BALB/c mice. We first analyzed trabecular and cortical bone mass by micro computed tomography (µCT) (**Figure 1**). The 3D reconstructed images of femurs showed that trabecular bone mass and structure in *J_H_^-/-^* mice was comparable to that observed in WT mice (**Figure 1A**). However, trabecular bone mass and structure in both λ5^-/-^ and *J_H_^-/-^* ${\lambda 5}^{-/-}$ mice was significantly decreased relative to both WT and *J_H_^-/-^* mice. Quantification of trabecular bone volume over total volume (BV/TV) showed a non-significant decrease in trabecular bone mass in *J_H_^-/-^* mice (**Figure 1B**). By contrast, the trabecular BV/TV was significantly decreased by 73% and 75% in the λ5^-/-^ *J_H_^-/-^* and ${\lambda 5}^{-/-}$ mice, respectively, when compared to WT controls. Trabecular numbers (Tb.N) in *J_H_^-/-^* mice were comparable to WT but were decreased by ~30% in λ5^-/-^ and *J_H_^-/-^* ${\lambda 5}^{-/-}$ mice (**Figure 1B**). The decrease in trabecular numbers in both λ5^-/-^ and *J_H_^-/-^* ${\lambda 5}^{-/-}$ mice caused a 39% and 40% increase, respectively, in trabecular spacing when compared to WT controls (**Figure 1B**). Trabecular thickness was significantly decreased in *J_H_^-/-^* ${\lambda 5}^{-/-}$ mice (**Figure 1B**).

Cortical bone sections were also examined in six-month-old WT, λ5^-/-^, *J_H_^-/-^*, and *J_H_^-/-^* ${\lambda 5}^{-/-}$ mice (**Figure 1C**). Cortical BV/TV quantification showed no difference among WT, *J_H_^-/-^* and λ5^-/-^ mice (**Figure 1D**). However, the combined absence of λ5 and post proB B cells in *J_H_^-/-^* ${\lambda 5}^{-/-}$ mice resulted in a significant decrease of 23% in cortical BV/TV when compared to WT mice. Together these results suggest varying contributions of λ5 and post proB cell populations in maintaining both trabecular and cortical bone mass in six-month-old adults.

### Bone turnover is increased upon deficiency of λ5 and/or post pro B cells

**Figure 2:**
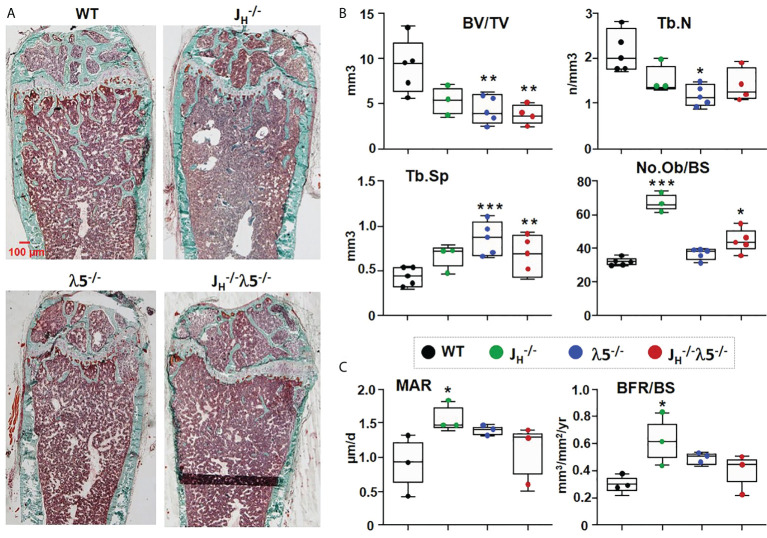
Osteoblast numbers and bone synthesis are increased in B cell-deficient mice. **(A)** Undecalcified femurs from 6‐month‐old females were embedded in plastic, sectioned laterally, and stained with Masson’s trichrome. Representative images are shown at 2X magnification. **(B)** Average histomorphometric values pooled from n=3-5 mice per group. Bone parameters shown are trabecular BV/TV ratio, Tb.N=trabecular number, Tb.Sp=trabecular space, and No.Ob/BS= osteoblast number per bone surface. **(C)** Dynamic histomorphometry was performed on calcein‐labeled bones. Average mineral apposition rate (MAR) and bone formation rate (BFR/BS) are shown. Statistical significance relative to WT mice was calculated by ANOVA. *p<0.05, **p<0.01, ***p<0.001. Scale bar: 100µm.

For a better understanding of possible factors leading to decreases in bone mass, we performed histomorphometric analyses of femoral bone in six-month old WT and mutant mice. Masson’s trichrome staining revealed an overall decrease in trabecular bone in the *J_H_^-/-^*, λ5^-/-^ and *J_H_^-/-^* ${\lambda 5}^{-/-}$ mice when compared to WT controls (**Figure 2A**).

Consistent with the prior µCT analysis, histomorphometric quantification showed a decrease in BV/TV. Compared to WT, *J_H_^-/-^* mice showed a 42% decrease, while λ5^-/-^ and *J_H_^-/-^* ${\lambda 5}^{-/-}$ mice showed a statistically significant 53% and 59% decrease, respectively, in BV/TV (**Figure 2B**). Similarly, trabecular numbers were decreased in *J_H_^-/-^* and *J_H_^-/-^* ${\lambda 5}^{-/-}$ mice, but the 45% decrease was statistically significant only in the λ5^-/-^ mice. The decrease in trabecular number led to a significant increase in trabecular spacing of 95% in λ5^-/-^ and 48% in *J_H_^-/-^* ${\lambda 5}^{-/-}$ mice (**Figure 2B**). Trabecular thickness was also decreased in all three of the mutant strains (data not shown). The number of osteoblasts per bone surface were significantly increased in both *J_H_^-/-^* and *J_H_^-/-^* ${\lambda 5}^{-/-}$ mice, but the increase in the λ5^-/-^ mice did not achieve statistical significance (**Figure 2B**). An assessment of dynamic bone synthesis revealed a significant increase in both the mineral apposition rate and bone formation rate in *J_H_^-/-^* mice (**Figure 2C**).

**Figure 3:**
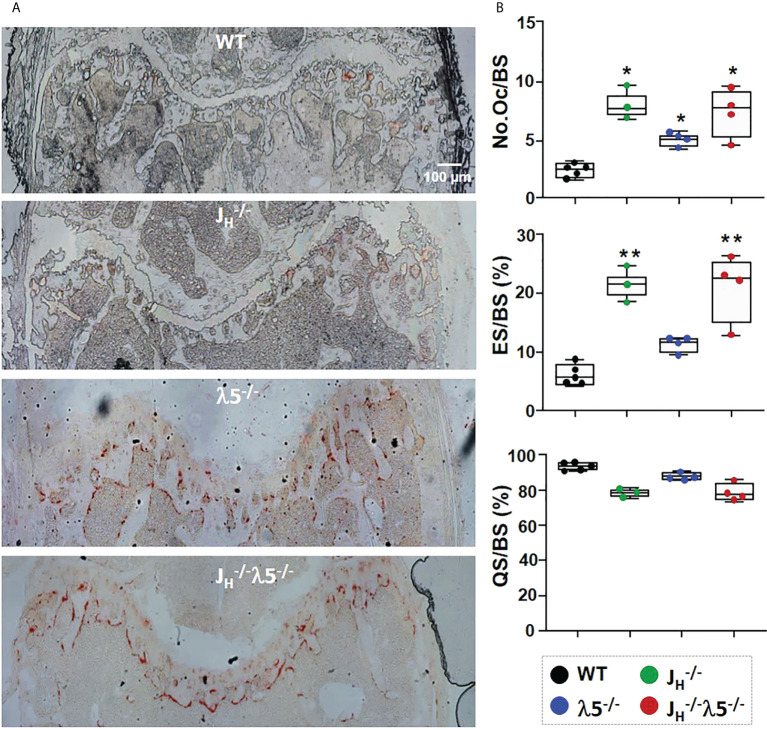
Increased numbers of osteoblasts and bone resorption upon loss of λ5 and/or post proB cells. **(A)** Representative images from six‐month old female femurs stained for TRAP activity. **(B)** Histomorphometric data pooled from n=3-5 mice per group show the average number of osteoclasts per bone surface (N.Oc/BS), erosion surface per bone surface (ES/BS) and the quiescent surface to bone surface (QS/BS). Statistical significance relative to WT mice was calculated by ANOVA. *p<0.05, **p<0.01. Scale bar: 100µm.

To examine the effect of the mutant alleles on bone resorption, sections of femur were stained for tartrate-resistant acid phosphatase (TRAP) to detect osteoclasts. Relative to WT controls, all mutants showed an overall increase in TRAP staining compared to WT mice (**Figure 3A**). Histomorphometric quantification showed a statistically significant 2-fold increase in the numbers of osteoclasts in λ5^-/-^ mice and a 3-fold increase in both *J_H_^-/-^* and *J_H_^-/-^* ${\lambda 5}^{-/-}$ mice (**Figure 3B**). The increase in the number of osteoclasts was accompanied by a ~3-fold increase in the erosion surface per bone surface in the *J_H_^-/-^* mice and *J_H_^-/-^* ${\lambda 5}^{-/-}$ mice. While an increase in the erosion surface in λ5^-/-^ mice was also noted, this change did not achieve statistical significance. Accordingly, the quiescent surface per bone surface showed a slight decrease in all mutant mice (**Figure 3B**), although again the effect was most prominent in mice lacking post proB cells. The increase in both the number of bone forming osteoblasts and bone resorbing osteoclasts point to an increase in bone turnover in the mice that lack post proB cells.

### λ5 deficiency accelerates age-associated trabecular bone loss

In order to evaluate the influence of λ5 on bone mass during homeostasis and aging, we used µCT to analyze female femoral bones at one-month of age, representing development of bone in young mice; at six-months of age, representing skeletal maturation; at twelve-months of age, representing bone homeostasis after skeletal maturation; and at twenty-two-months of age, representing bone aging.

**Figure 4:**
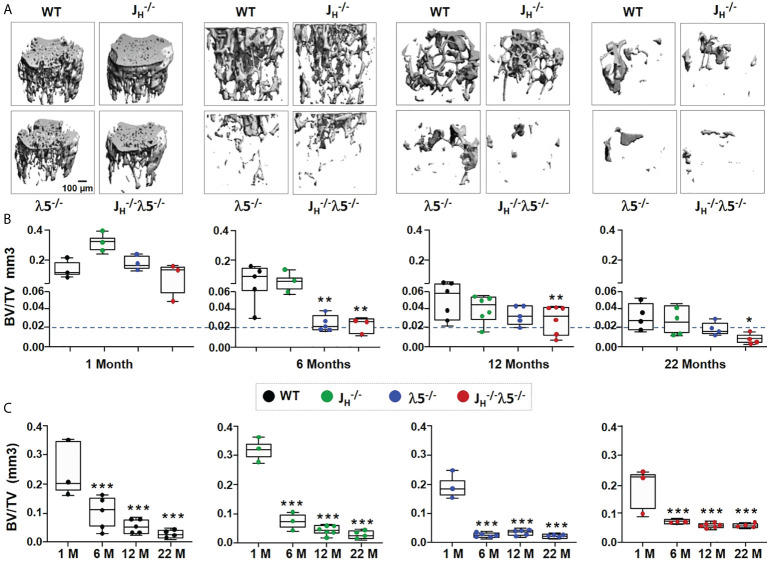
Absence of λ5 accelerates age-associated trabecular bone loss. **(A)** Representative 3D reconstruction of µCT images of trabecular bone from femurs of WT, *J_H_^-/-^*, λ5^-/-^ and *J_H_^-/-^* λ5^-/-^ female mice at one-, six-, twelve- and twenty-two-months of age are shown. **(B)** Graph shows quantification of trabecular BV/TV during post-natal development for indicated genotype. The blue dashed line represents the level of trabecular BV/TV of six-month old λ5^-/-^ mice compared to other genotypes. **(C)** The relative ratio of trabecular BV/TV of WT, *J_H_^-/-^*, λ5^-/-^ and *J_H_^-/-^* λ5^-/-^ mice at one-, six-, twelve- and twenty-two-months of age. Data were obtained from n=3-6 mice per group. Statistical significance relative to WT mice was calculated by ANOVA. *p<0.05, **p<0.01, ***p<0.001. Scale bar: 100µm.

Starting from six-months of age, λ5^-/-^ and *J_H_^-/-^* ${\lambda 5}^{-/-}$ mice had decreased trabecular bone mass when compared to WT and *J_H_^-/-^* strains (**Figure 4A**). The ratio of trabecular BV/TV at one-month of age in all mutant mice was comparable to WT controls (**Figure 4B**). At six-months of age, the trabecular BV/TV of *J_H_^-/-^* mice was equivalent to WT mice. However, both λ5^-/-^ and *J_H_^-/-^* ${\lambda 5}^{-/-}$ mice showed a significant decrease in BV/TV relative to WT controls (**Figure 4B**). The significant decrease in BV/TV in *J_H_^-/-^* ${\lambda 5}^{-/-}$ mice was also apparent at twelve- and twenty-two-months of age (68% and 77%, respectively). At these ages, the decrease in BV/TV of *J_H_^-/-^* ${\lambda 5}^{-/-}$ relative to *J_H_^-/-^* mice also remained statistically significant (61% and 75%, respectively). Stikingly, the BV/TV of six-month old λ5^-/-^ and *J_H_^-/-^* ${\lambda 5}^{-/-}$ mice was similar to or even lower than that of twenty-two-month old WT and *J_H_^-/-^* mice, suggesting accelerated trabecular bone loss (**Figure 4B**, dashed line).

We next analyzed changes in the trabecular bone mass in each genotype during skeletal aging (**Figure 4C**). Compared to one month, WT and *J_H_^-/-^* mice showed a stepwise gradual decrease in trabecular BV/TV from six-months to twenty-two-months. By contrast, the λ5^-/-^ and *J_H_^-/-^* λ5^-/-^ strains, which at six months already had a very low trabecular BV/TV, showed no significant further decline in trabecular bone mass from six-months to twenty-two-months. Thus, λ5^-/-^ and *J_H_^-/-^* λ5^-/-^ mice reach an early plateau in trabecular bone loss starting at six-months of age. Together, these data indicate that the absence of λ5 affects trabecular bone mass over time by promoting accelerated trabecular bone loss similar to the effects seen with aging.

### The combined loss of λ5 and early/immature/mature B cells delays postnatal acquisition of cortical bone mass

**Figure 5:**
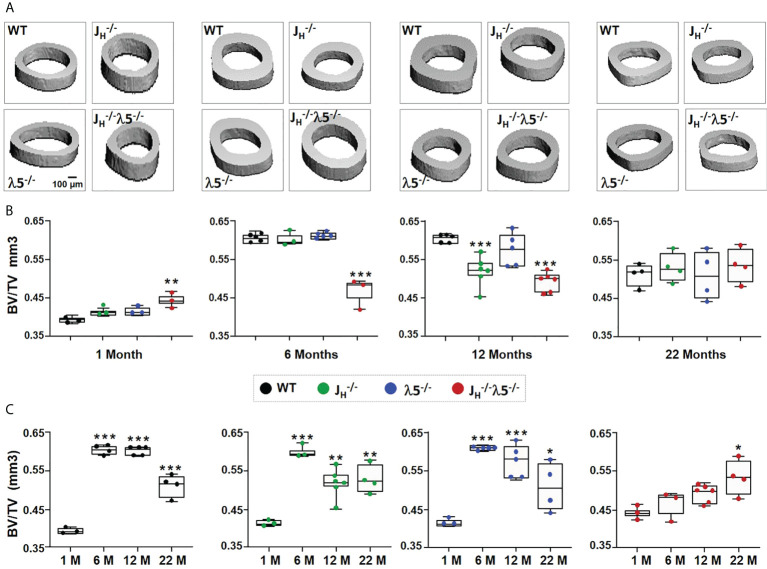
Age-related changes in cortical bone mass in the absence of λ5, post proB cells or both. **(A)** Representative 3D reconstruction of µCT of cortical bone from femurs of WT, *J_H_^-/-^, *λ5^-/-^ and *J_H_^-/-^* λ5^-/-^ mice at 1, 6, 12 and 22 months of age. **(B)** The box and whisker graph shows quantification of cortical BV/TV for indicated genotypes at one-, six-, and twelve- and twenty-two-months of age. **(C)** The relative ratio of cortical BV/TV of WT, *J_H_^-/-^*, λ5^-/-^ and *J_H_^-/-^* λ5^-/-^ mice at the indicated ages. Data were obtained from n=3-6 mice per group. Statistical significance was calculated by ANOVA. *p<0.05, **p<0.01, ***p<0.001. Scale bar: 100µm.

In contrast to trabecular bone, cortical bone mass showed variable acquisition during development and aging among the studied genotypes (**Figure 5A**). Acquisition of cortical bone mass BV/TV from one- to six-months showed an increase of 34% in WT, and 31% in *J_H_^-/-^* and λ5^-/-^ mice. Cortical bone mass in WT, *J_H_^-/-^* and λ5^-/-^ mice rose from one-month to a peak at six-months of age, and then declined over the following sixteen months (**Figure 5B**). In contrast, *J_H_^-/-^* λ5^-/-^ mice did not demonstrate the dramatic increase in cortical bone mass observed at six-months of age in the other three genotypes. During postnatal development and skeletal maturation, cortical BV/TV of six-month old *J_H_^-/-^* λ5^-/-^ mice was significantly (23%) less than WT, *J_H_^-/-^* and λ5^-/-^ mice. Minor differences in cortical BV/TV of all genotypes at twenty-two months were statistically indistinguishable. Thus, peak cortical bone mass in *J_H_^-/-^* λ5^-/-^ mice was achieved at twenty-two-months, and then only at a level equivalent to the aging-associated decrease in aged cortical bone mass observed in the other three genotypes.

The pattern of cortical bone mass acquisition and loss as a function of age differed for *J_H_^-/-^* λ5^-/-^ mice when compared to all the other studied strains (**Figure 5C**). The normal increase in cortical bone mass from one to six-months that was then followed by a decline at twelve- or twenty-two-months, which was observed in both WT and single mutant strains ( *J_H_^-/-^* and λ5^-/-^), was not observed in the double knockout mutant. Instead, there was a slow stepwise increase in the BV/TV of *J_H_^-/-^* λ5^-/-^ mice at one-, six-, and twelve- months of age that then peaked at twenty-two-months of age. This suggests that the process of acquisition of peak cortical bone mass relies on the joint presence of both λ5 and post proB B cells. Our data also indicate that both λ5 and post proB cells are needed to maintain peak cortical bone mass during skeletal aging.

### Adoptive transfer of bone marrow cells expressing λ5 regenerates adult bone mass

To investigate the role of λ5 in dynamic bone synthesis we perfomed adotive transfer experiments. Recipient three-month old *J_H_^-/-^* λ5^-/-^ or WT mice were irradiated with two doses of 600 rads each. This radiation dose eliminates all developing hematopoietic progenitors and ensures that only mice with successful marrow engraftment will survive (33, 34). The bone marrow niche of the irradiated mice was reconstituted by intravenous transfer of whole bone marrow cells from WT (λ5-sufficient) or *J_H_^-/-^* λ5^-/-^ (λ5-deficient) mice. To allow proper engraftment of transferred marrow cells and assess their capacity to synthetize bone, the recipient mice were maintained for six months after irradiation. This time frame was chosen based on our finding that trabecular bone mass is significantly decreased in *J_H_^-/-^* λ5^-/-^ mice over a six-month period (**Figures 1**, **4**). At six months post-transfer, femurs were analyzed by both histomorphometry and µCT.

**Figure 6:**
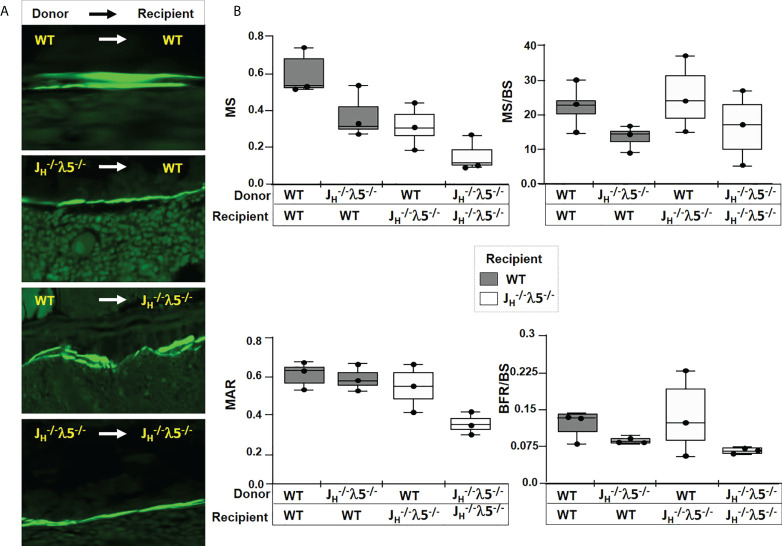
Dynamic bone synthesis upon adoptive transfer of λ5-sufficient bone marrow cells. **(A)** Representative images of calcein labelled new bone synthesis in irradiated mice reconstituted with bone marrow from the indicated genotypes. Femur endosteal surfaces were analyzed 6-months after reconstitution. **(B)** Quantification of mineralized surface (MS), ratio of mineralized surface over bone surface (MS/BS), mineral apposition rate (MAR), and bone formation rate over bone surface (BFR/BS). The genotypes of the donor and the recipient mice are indicated. Data were obtained from n=3-5 mice per group. The experimental data are the average of three repeats. Statistical significance was calculated by ANOVA.

To evaluate dynamic bone formation, the bone marrow recipient mice were double injected with calcein at seven-days and two-days prior to sacrifice and harvest of the skeletal tissue. Calcein is a fluorescent dye that is incorporated into synthesized bone and the two staggered doses can distinguish new bone synthesis over a defined timeframe. Imaging of femur sections revealed a successful reconstitution of the bone marrow and active bone formation at the endosteal surface of the cortical bone in WT mice transplanted with WT bone marrow (**Figure 6A**). In contrast, calcein positive cortical bone surfaces appeared to be decreased in WT mice receiving λ5-deficient marrow cells. *J_H_^-/-^* λ5^-/-^ mice transplanted with the λ5-deficient marrow cells also appeared to demonstrate a diminished capacity for bone synthesis whereas transfer of WT marrow cells (λ5-sufficient) appeared to rescue the decreased bone formation in the *J_H_^-/-^* λ5^-/-^ mice (**Figure 6A**).

We also noted interesting trends after histomorphometric quantification of the dynamic bone parameters in WT mice that received the λ5-deficient marrow cells. There was a decrease in mineralized surface (MS), with a decrease in mineralized surface over bone surface (MS/BS) (**Figure 6B**). There was also a decrease in the mineral apposition rate (MAR) and in the bone formation rate over bone surface (BFR/BS). Conversely, transplant of λ5-sufficient WT marrow cells into the *J_H_^-/-^* λ5^-/-^ mice appeared to lead to an increase in MS and MS/BS, when compared to *J_H_^-/-^* λ5^-/-^ mice that received λ5-defeicient marrow cells. Consistent with these observations, an increase in MAR, and BFR/BS was noted in *J_H_^-/-^* λ5^-/-^ mice receiving λ5-sufficient marrow cells (**Figure 6B**). These data show a trend of reversibility of cortical bone loss when WT marrow is transplanted into irradiated *J_H_^-/-^* λ5^-/-^ mice and support the view that λ5-expressing cells and post proB cells are required for postnatal bone formation. However, these differences did not achieve statistical significance and thus we view them with caution.

### Transfer of λ5-deficient bone marrow cells induced bone loss.

**Figure 7:**
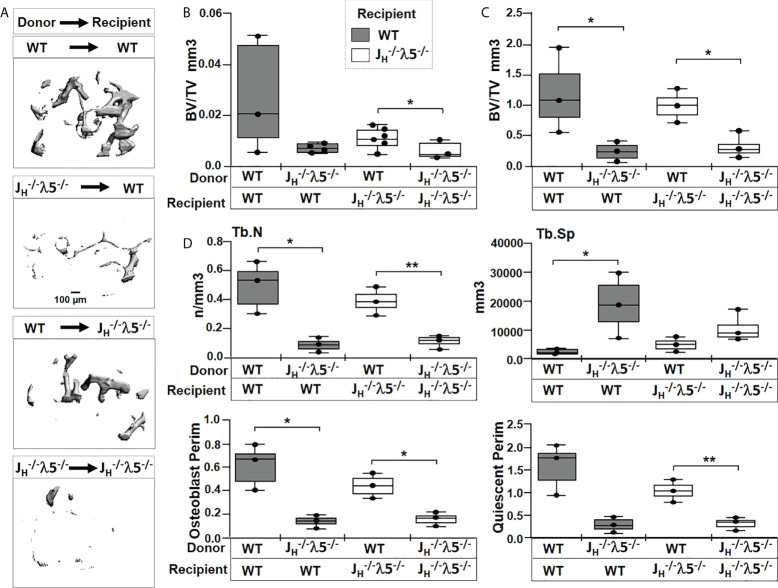
Transfer of λ5-sufficient whole marrow cells rescue trabecular bone in *J_H_^-/-^* λ5^-/-^ mice. **(A)** Representative µCT images of trabecular bone in femurs of radio-ablated WT or *J_H_^-/-^* λ5^-/-^ females reconstituted with *J_H_^-/-^* λ5^-/-^ or WT whole bone marrow cells. Femurs were analyzed six months after marrow reconstitution. **(B)** Quantification of the bone volume over total volume (BV/TV) from µCT analysis, and **(C)** from histomorphometric analysis of the same femur after histologic processing. **(D)** Selected bone parameters quantified from the histomorphometric analysis of indicated genotypes are shown in bar graphs. Trabecular numbers (Tb.N); Trabecular spacing (Tb.SP); Osteoblast Perimeter (Osteoblast Perim), Quiescent Perimeter (Quiescent Perim). Data were obtained from n=3-5 mice per group. The experimental data are the average of three repeats. Statistical significance was calculated by ANOVA. *p<0.05, **p<0.01. Scale bar: 100µm.

Mice lacking either λ5 or post pro B cells, or both, showed increased turnover of trabecular bone mass during aging (**Figures 1** **-** **4**). µCT analysis showed that transfer of *J_H_^-/-^* λ5^-/-^ bone marrow into irradiated WT recipients led to a 73% decrease in trabecular BV/TV, compared to WT mice that received WT bone marrow cells (**Figures 7A, B**). WT mice receiving *J_H_^-/-^* λ5^-/-^ bone marrow also showed a 14% decrease in trabecular numbers and a concomitant 11% increase in trabecular spacing (data not shown). In contrast, *J_H_^-/-^* λ5^-/-^ mice that received WT bone marrow cells showed a 53% increase in trabecular BV/TV, compared to the mutant mice transplanted with *J_H_^-/-^* λ5^-/-^ bone marrow cells (**Figures 7A, B**). In these mice, we also noted a 13% increase in trabecular number leading to a 14% decrease in trabecular spacing (data not shown). Thus, although the transplantation studies did not yield statistically significant differences in cortical bone homeostasis, the studies of trabecular bone formation did, providing further evidence that cortical and trabecular bone are regulated differently.

To better quantify contribution of λ5 in bone synthesis, histomorphometric analysis were performed on Masson’s trichrome stained femurs (**Figures 7C, D** and **Supplementary Figure S3**). The transfer of λ5-deficient marrow into irradiated WT mice showed an 80% decrease in trabecular BV/TV compared to WT mice that received λ5-sufficient marrow (**Figure 7C**). We also noted a decrease in trabecular bone surface (BS) and the ratio of bone surface over total volume (BS/TV) (data not shown). In contrast, irradiated *J_H_^-/-^* λ5^-/-^ mice receiving WT bone marrow showed a 66% increase in trabecular BV/TV relative to mice receiving λ5-deficient marrow cells (**Figure 7C**). The *J_H_^-/-^* λ5^-/-^ recipients of WT marrow cells also showed a 68% increase in bone surface and a 72% increase in BS/TV (data not shown). The histomorphometric data showing rescue of trabecular bone formation in *J_H_^-/-^* λ5^-/-^ mice by λ5-sufficient marrow are consistent with the µCT analysis.

Evaluation of additional bone parameters in the irradiated WT mice receiving *J_H_^-/-^* λ5^-/-^ bone marrow uncovered a 82% decrease in trabecular numbers and an 88% increase in trabecular spacing (**Figure 7D**). In addition, a decrease in osteoblast perimeter (80%) and quiescent perimeter (81%) relative to irradiated WT mice receiving WT bone marrow was observed. The rescue of irradiated *J_H_^-/-^* λ5^-/-^ mice with the WT bone marrow resulted in a 72% increase in trabecular number with a corresponding 56% decrease in trabecular spacing. These mice also showed a 67% increase in osteoblast perimeter and a 68% increase in quiescent perimeter.

We also evaluated the numbers of osteoblasts and osteoclasts in the adoptively transfered mice. Histomorphometric quantification revealed a trend for an increase in the numbers of osteoblasts per bone surface, osteoblast surface, numbers of osteoclasts per bone surface, and osteoclast surface in WT mice that received *J_H_^-/-^* λ5^-/-^ bone marrow (**Supplementary Figure S3B**). However, while these findings are consistent with the increase in osteoblasts and osteoclasts, and thus bone turnover, previously observed in six-month old non-transplanted *J_H_^-/-^* λ5^-/-^ mice (**Figures 2**, **3**), they did not reach statistical significance.

## Discussion

The appearance of immunoglobulin bearing B cells during evolution coincides with the appearance of osteoblasts and osteoclasts, the cells involved in bone formation and resorption, respectively (35). In mice and humans, progenitor B cells and bone cells develop in environmental niches that are adjacent to each other on the endosteal surface of bone. Cortical and trabecular bone have different structure and metabolic features and there is physical interaction with B lineage cells in the bone marrow. This close apposition creates an environment where secreted factors, direct cell-to-cell contacts, or both, could influence bone homeostasis. In this intimate environment, bone cells and their stromal progenitors provide growth signals and cytokines that promote the differentiation and growth of progenitor B cells (36). In turn, B lineage cells, in particular mature B cells and plasma cells, have been shown to produce modulators that influence bone formation and resorption (37).

Among the factors known to be involved in B cell and bone crosstalk are receptor activator of nuclear factor kappa-B (RANK), RANK ligand (RANKL) and osteoprotegerin (OPG) (37–40). Mature B cells can produce RANKL and OPG. RANKL bound to RANK on the surface of osteoclast precursors promotes bone resorption. OPG can compete with RANKL for RANK binding, impairing RANK activity and thus reducing osteoclast activity. The balance between OPG and RANKL activity is thus considered a major factor controlling bone remodeling. Loss of mature B cells impairs OPG/RANKL ratio leading to decreased cortical bone in four-month old µMT^-/-^ C57BL/6 mice (41). Blocking B cell development at an early stage with Pax5 deletion leads to an early death with severe osteopenia due to an increase in the number of osteoclasts (26). However, a change in bone phenotype was not observed in very young RAG-1^-/-^ or μMT^-/-^ mice, both of which exhibit a block in B cell development at progenitor stages (26). These conflicting studies left open the question of the role of progenitor B cells in regulating postnatal bone development.

λ5 is a key component of the surrogate light chain and the preB cell receptor (preBCR). In the absence of λ5, preB cells cannot express a preBCR, blocking early B cell development. However, mature B cell compartments are filled over time. In six-month old λ5^-/-^ and μMT^-/-^ female C57BL/6 mice, we previously found that both trabecular BV/TV and cortical BV/TV were reduced. The reduction in bone mass in μMT^-/-^ mice was in accordance with the prevailing view that mature B cells and plasma cells influence bone homeostasis through their contribution to the RANKL/RANK/OPG axis. However, the reduction in bone mass in the λ5^-/-^ mice, which unlike μMT^-/-^ mice contain both mature B cells and plasma cells, suggested that λ5 pathways independent of the RANKL/RANK/OPG mature B cell axis might also be playing a major role in acquisition, maintenance, and/or resorption of bone mass (6).

In order to gain more insight and to test for possible strain differences, here we studied the effect of the absence of λ5 (λ5^-/-^), immunoglobulin H chains ( *J_H_^-/-^*) or both (*J_H_^-/-^* λ5^-/-^) on a BALB/c background. While the general outlines of bone and B cell biology are very similar in C57BL/6 and BALB/c, there are notable differences. In both strains, proB and preB cell numbers and λ5 expression decrease with advancing age (23). However, while BALB/c mice begin with similar numbers of proB cells as C57BL/6, they have more than three times as many preB cells (41). Thus, when compared to BALB/c, C57BL/6 mice are relatively deficient in λ5 and preBCR expression. Bone mass in BALB/c reaches peak levels earlier than C57BL/6 (42). BALB/c mice exhibit greater trabecular numbers and thickness and they have stiffer and harder trabecular bone than C57BL/6 mice. In contrast, cortical bone in C57BL/6 mice is considered to be harder than in BALB/c (43).

In six-month old female BALB/c mice, we found that absence of λ5 resulted in a decreased ratio of bone volume to total volume in the trabecular bone and in a decrease in trabecular number with a corresponding increase in trabecular spacing. Since these findings are consistent with our previous observations in C57BL/6 mice (6), we may thus conclude that λ5 expression is crucial for proper development of trabecular bone at six-months of age in female mice, irrespective of strain.

However, at six-months of age there were significant differences in cortical bone between λ5^-/-^ C57BL/6 and BALB/c mice. Unlike λ5^-/-^ C57BL/6 mice, which showed a decrease in cortical bone mass, λ5^-/-^ BALB/c mice had no change in cortical BV/TV. Moreover, *J_H_^-/-^* BALB/c mice showed no change in either cortical or trabecular BV/TV, suggesting that at six-months of age that there was a decoupling between the role of µHC and B lineage cells past the preBCR cell stage, and the role of λ5 in bone formation. To test the extent of decoupling between µHC and λ5 pathways, we generated *J_H_^-/-^* λ5^-/-^ mice. Loss of trabecular bone in λ5^-/-^ deficient BALB/c mice did not vary with the presence or the absence of µHC. We conclude that the pathway by which λ5 exerts its effect on the loss of trabecular bone in six-month old BALB/c mice is independent of the role of µHC and thus is likely not dependent on preBCR activation.

Conversely, the loss of cortical bone mass in *J_H_^-/-^* λ5^-/-^ mice in the context of normal cortical bone mass in either λ5^-/-^ or ${\text{J}}_{-/-}^{\text{H}}$ mice suggests that the µHC- and λ5-dependent pathways can compensate for the loss of either one of these pathways, but that the absence of both pathways adversely affects cortical bone mass in BALB/c mice.

As noted above, λ5^-/-^ mice have low numbers of late preB cells and immature B cells but can produce late preB cells bearing an IgM B cell receptor and have mature B cells; and *J_H_^-/-^* mice lack preB and mature B cells but contain progenitor B cells that express λ5. Loss of both components in *J_H_^-/-^* λ5^-/-^ mice leads to a complete loss of expression of µHC and λ5, a complete loss of preBCR expression, and a complete loss of B cells past the proB cell stage.

The block in B cell development in λ5^-/-^ mice is leaky (7, 44). One of us (PDB) previously showed that some proB cells rearrange their light chain genes before their heavy chain genes, bypassing selection by the surrogate light chain (8). In other cases, a µH chain alone can generate a signal sufficient to allow passage through the preB cell checkpoint (45). As a result, λ5^-/-^ mice produce preB, immature B, and mature B cells. Moreover, once the preBCR checkpoint has been passed, λ5 expression ceases. There is no known difference in function between a B cell that develops in a λ5^-/-^ mouse versus a B cell that develops in a WT mouse.

Over time, the progeny of cells that managed to bypass the preBCR checkpoint accumulate and by six months of age the numbers of mature B cells approach wild type mature B cell numbers (6, 7). At this age, six months of age, we find that λ5-deficient mice, which have mature B cells in the periphery and in the bone marrow, demonstrate decreased trabecular bone mass (**Figure 1**). Since immature B cells and mature B cells are present in these λ5-deficient mice, this experimental result is the basis of our conclusion that the loss of bone in λ5^-/-^ mice reflects the loss of λ5 expression and not the absence of B cells. Corroboration of this conclusion comes from study of *J_H_^-/-^* mice that express λ5 but are unable to produce preB cells, immature B cells and mature B cells. We found no significant difference from WT in trabecular bone mass in these mice.

To summarize, the experiments reported in this manuscript show that trabecular bone mass is diminished in the absence of λ5, but not in the absence of preB, immature B and mature B cells.

The individual functions of λ5 and μHC intersect in the transition from the early to late preB cell stage in their actions as components of the preB cell receptor. Thus it is possible that normal development of cortical bone in BALB/c mice in particular depends on λ5-influenced preBCR expression, function or signal transduction. Galectin-1 (Gal1) interacts directly with λ5 and functions as a stromal cell ligand for the preBCR (46). λ5-GAL1 binding leads to pre-BCR clustering into a Gal1-counter-receptor complex lattice that is polarized at the preB-stromal cell-synaptic junction, providing a possible mechanism by which the preBCR could influence interactions between preB cells and stromal cells, which in turn could influence bone growth. Whether there is a role for soluble λ5-Gal1 function in bone development is unknown. Dissection of these pathways is a current focus of our laboratories.

Both uCT and histomorphometry reveal a decline in BV/TV in λ5^-/-^ and *J_H_^-/-^* λ5^-/-^ mice. A decrease was also observed in *J_H_^-/-^* mice, but the decrease was not significant. These findings suggest abrogation and/or a reduction in signalling through the preBCR in late preB cells and/or through the BCR in immature B cells contributes to the decrease in bone mass seen in all three strains by differentially influencing osteoblast and osteoclast numbers with downstream effects on bone turnover activity. *J_H_^-/-^* mice demonstrate the greatest relative increase in osteoblast versus osteoclast numbers. The balance between the gain of osteoblasts versus osteoclasts is weighted in favor of the osteoclasts in λ5^-/-^ and *J_H_^-/-^* λ5^-/-^ mice. λ5^-/-^ mice demonstrate a limited effect on osteoblast numbers but a significant increase in osteoclast numbers. *J_H_^-/-^* λ5^-/-^ mice have a higher number of osteoblasts than λ5^-/-^ mice, but they also have an increase in osteclasts that is similar to *J_H_^-/-^* deficiency alone. These differences in the balance between osteoblast and osteoclast numbers could explain why *J_H_^-/-^* mice exhibit the smallest decline in bone mass.

Previous studies by others have shown that aging is associated with a decline in λ5 expression by both proB and preB cells (47). This led us to further hypothesize that the effect of λ5 on bone might vary as a function of age, and that changes in λ5 expression might be associated with changes in bone mass. As noted above, quantitative and temporal changes in λ5 expression between C57BL/6 and BALB/c mice might contribute to some, if not all, of the differences in bone mass that we see in our mutant mice. We thus extended our observations in the BALB/c strain to include one-, twelve- and twenty-two-months old mice. We found that aging in females had a profound effect on the roles of λ5 and µHC in acquiring and maintaining both trabecular and cortical bone mass.

In trabecular bone, loss of λ5 either alone (λ5^-/-^) or combined with loss of µHC ( *J_H_^-/-^* λ5^-/-^) led to an inability to maintain trabecular bone mass at a time after one-month of age and before six-months of age. Notably, in the absence of λ5, trabecular bone mass at six-months of age was lower than trabecular bone mass in WT mice at twenty-two-months of age. These findings suggest that loss of λ5, either in the presence or absence of µHC, leads to accelerated trabecular bone loss over time yielding a phenotype consistent with premature aging of the bone.

A strikingly different pattern was observed in cortical bone. Unlike trabecular bone, where bone mass was greatest at one-month of age in the WT mice and declined thereafter, acquisition of cortical bone mass occurs between one-month and six-months of age, stabilizes between 6 and twelve-months, and then suffers a decrease between twelve- and twenty-two-months of age. At six-months of age, loss of either λ5 or µHC had no statistically significant effect on cortical bone mass when compared to WT. Mice lacking λ5 also had slightly lower cortical bone mass at twelve-months than WT mice, but this difference did not achieve statistical significance.

Doubly deficient *J_H_^-/-^* λ5^-/-^ mice completely failed to acquire normal cortical bone volume by six-months or twelve-months. At twenty-two-months, doubly deficient *J_H_^-/-^* λ5^-/-^ mice had slightly higher cortical bone volume than singly deficient λ5^-/-^ or *J_H_^-/-^* mice, or WT mice. However, this occurred in the context of a lowering of bone volume in the singly deficient λ5^-/-^ or *J_H_^-/-^* mice, and the WT mice. These findings indicate that cortical bone acquisition and maintenance between one-month and twelve-months of age is dependent on the collaborative effect of both λ5 and immunoglobulin-expressing post proB B lineage cells, including cells involved in modulating the RANKL/RANK/OPG axis. Further, these findings also indicate that the means by which λ5 and immunoglobulin-expressing post proB B lineage cells are influencing acquisition and maintenance of bone during aging differs between trabecular and cortical bone.

A key issue in the osteology of aging, especially among women, is whether and how loss of bone can be ameliorated or even reversed.

Transfer of whole bone marrow cells from the λ5^-/-^ *J_H_^-/-^* mice, which lack all post-proB cells and λ5, into irradiated WT mice led to the loss of trabecular bone volume; whereas transfer of whole bone marrow from the WT mice led to an increase in trabecular bone volume. This process was associated with increased osteoblast activity. The observed quite striking bone phenotype can thus be attributed to B lineage cells and/or λ5, one of the major conclusions of the studies reported in this paper. Given that λ5 and progenitor B lineage cells expressing λ5 decrease with age, this raises the possibility that quantitative manipulation of the pathways associated with λ5 production and function at the proB or preB cell stage during aging could positively affect bone health.

We transferred whole bone marrow in order to include both hematopoietic progenitors and other bone marrow cellular components. This allowed us to reconstitute a complete donor bone marrow microenvironment, which was most likely to favor survival post irradiation and transplantation. In future studies we plan to perform more precise analyses of the specific cell subsets that are controlling bone volume. Future studies, which will include transplanation of bone marrow from λ5^-/-^ and *J_H_^-/-^* mice to dissect the specific role of λ5 versus other B cell subsets in bone biology, are an area of active investigation in our laboratories.

Among findings reported in this paper, we show that a preB cell protein, λ5, regulates trabecular bone homeostasis during aging independent of the presence or absence of preB, immature B, or mature B cells. Thus, we show that an early B-lineage molecule, λ5, is influencing trabecular bone formation outside of the previously described RANKL-associated pathways active in mature B cells. Therefore, we have identified a molecule whose presence or absence influences bone mass as a function of age. We are in process of elucidating molecular pathways of λ5 influence on bone, including testing the role of known λ5 ligands.

## Data availability statement

The original contributions presented in the study are included in the article/**Supplementary Material**. Further inquiries can be directed to the corresponding authors.

## Author contributions

Study design: MK, AJ and HS. Study conduct: MK and AJ. Data collection: MK, HR, and AJ. Data analysis: MK, AJ and HS. Data interpretation: MK, AJ and HS. Drafting manuscript: MK, PB, AJ and HS. All authors contributed to the article and approved the submitted version.

## Funding

Research reported in this publication was supported by the National Institute of Allergy and Infectious Diseases (HS), the National Institute of Arthritis and Musculoskeletal and Skin Diseases (AJ), the National Institute on Aging (AJ and MK), and the National Institute of Dental and Craniofacial Research (HR and MK) of the National Institutes of Health under award numbers R21AI134027 (HS), R01AR062091 (AJ), R56AG065129 (AJ), R56AG076730 (MK), and T90DE022736 (HR and MK). Support was also provided by a pilot grant from UAB GC-CODED. The content is solely the responsibility of the authors and does not necessarily represent the official views of the National Institutes of Health.

## Acknowledgments

Power analysis and statistics were performed with the help of Dr. David Redden Professor of Biostatistics at the UAB School of Public Health.

## Conflict of interest

The authors declare that the research was conducted in the absence of any commercial or financial relationships that could be construed as a potential conflict of interest.

## Publisher’s note

All claims expressed in this article are solely those of the authors and do not necessarily represent those of their affiliated organizations, or those of the publisher, the editors and the reviewers. Any product that may be evaluated in this article, or claim that may be made by its manufacturer, is not guaranteed or endorsed by the publisher.

## Supplementary material

Supplementary Figure 1 Stages of B cell development in the bone marrow and periphery and impairment of B cell development in the mutant mice used in this study. B cells develop in the bone marrow and can be categorized as Hardy (48) fractions **(A–E)**. B cells mature in the periphery (Transitional and Mature B cells). Expression of λ5 begins at Hardy fraction **(B)**, µHC appears at **(C)** but cannot be formed in the *J_H_^-/-^* mice due to the failure of VDJ_H_ rearrangement. Surface preBCR expression, composed of µHC and SLC (λ5 plus VpreB), appears at C’. The stage and completeness of the blocks in B cell development in the absence of λ5 and/or J_H_ are shown at the top of the figure. The dashed line represents a partial block in B cell development.

Supplementary Figure 2 Mouse genotyping and confirmation of the absence of preBCR expression in λ5^-/-^ mice. **(A)** Genotyping. The λ5 gene (*Igll1*) was amplified by specific primers. The presence of *Igll1* was confirmed in WT and *J_H_^-/-^* mice (lane1 and 2, red box) while its absence was confirmed in λ5^-/-^ and *J_H_^-/-^* λ5^-/-^ mice (lanes 3 and 4). **(B)** Flow Cytometry. Flow cytometry was used to confirm the absence of preBCR expression in the mutant λ5- deficient strains. Early B cell subsets including preB cells were identified based on expression of surface markers including: CD19, B220, AA4.1, and BP-1. Cells were fixed and permeabilized for intracellular staining. Antibodies specific for the preBCR (SL156 clone) and Mu HC were used to confirm the presence of the preBCR-expressing cells in WT mice (31.1% of cells) and their absence both on the cell surface and intracellularly in λ5^-/-^ mice.

Supplementary Figure 3 Comparable numbers of osteoblasts and osteoclasts in bone marrow of recipient mice. **(A)** Undecalcified femurs from adoptive transfer mice were embedded in plastic, sectioned and stained with Masson’s trichrome. Representative images from the endosteal surface are shown at 10X magnification. The yellow boxed region is shown at 40X magnification. **(B)** Average histomorphometric values pooled from the indicated recipient mice (n=3-5 mice per group). No.Ob/BS=Number of osteoblasts per bone surface, ObS/BS=Osteoblast surface per bone surface, No.Oc/BS=number of osteoclasts per bone surface and OcS/Bs=Osteoclast surface per bone surface. Statistical significance was calculated by AOVA. Scale bar: 100µm. Data were obtained from n=3-5 mice per group.
