# Corrigendum: Loss of early B cell protein λ5 decreases bone mass and accelerates skeletal aging

**DOI:** 10.3389/fimmu.2022.1114732

**Published:** 2023-02-13

**Authors:** Mohamed Khass, Harunur Rashid, Peter D. Burrows, Amjad Javed, Harry W. Schroeder

**Affiliations:** ^1^ Department of Medicine, University of Alabama at Birmingham, Birmingham, AL, United States; ^2^ Department of Oral and Maxillofacial Surgery, University of Alabama at Birmingham, Birmingham, AL, United States; ^3^ Department of Microbiology, University of Alabama at Birmingham, Birmingham, AL, United States

**Keywords:** λ5, B cells, bone mass, skeletal aging, Trabecular bone recovery

In the published article [Reference number (4) in the **Discussion** section at the sentence that reads “The balance between OPG and RANKL activity is thus considered a major factor controlling bone remodeling. Loss of mature B cells impairs OPG/RANKL ratio leading to decreased cortical bone in four-month old µMT-/- C57BL/6 mice (4)”. This was incorrect and missing the correct reference and it should be changed to a new reference (B cells and T cells are critical for the preservation of bone homeostasis and attainment of peak bone mass *in vivo*. Blood. 2007 May 1; 109(9): 3839–3848. doi: 10.1182/blood-2006-07-037994. PMCID: PMC1874582 PMID: 17202317)]. The citation has now been inserted in the **Discussion** section, and the paragraph should read:

“The balance between OPG and RANKL activity is thus considered a major factor controlling bone remodeling. Loss of mature B cells impairs OPG/RANKL ratio leading to decreased cortical bone in four-month old µMT-/- C57BL/6 mice (41)”.

In the published article, the reference for (25) in the introduction section at the sentence that reads “JH^-/-^ B lineage cells lack all four JH gene segments, cannot produce immunoglobulin HC proteins, and thus cannot associate with SLC even in their secretory form (25)” was incorrect as “Glatt V, Canalis E, Stadmeyer L, Bouxsein ML. Age-related changes in trabecular architecture differ in female and male C57BL/6J mice. J Bone Miner Res (2007) 22(8):1197–207. doi: 10.1359/jbmr.070507”. It should be reference “Gu H, Zou YR, Rajewsky K. Independent control of immunoglobulin switch recombination at individual switch regions evidenced through cre-loxP-mediated gene targeting. Cell (1993) 73(6):1155–64. doi: 10.1016/0092-8674(93)90644-6”. Thus the correct sentence in the manuscript should be now

“J_H_^-/-^ B lineage cells lack all four J_H_ gene segments, cannot produce immunoglobulin HC proteins, and thus cannot associate with SLC even in their secretory form (25)”

In the published article, the reference (9) in the method section at the sentence that reads “The generation of JH-/- and λ5-/- mice on a C57BL/6 background has been previously reported (7, 9)” was incorrect as “Kitamura D, Roes J, Kuhn R, Rajewsky K. A b cell-deficient mouse by targeted disruption of the membrane exon of the immunoglobulin mu chain gene. Nature (1991) 350(6317):423–6. doi: 10.1038/350423a”. It should be reference “Gu H, Zou YR, Rajewsky K. Independent control of immunoglobulin switch recombination at individual switch regions evidenced through cre-loxP-mediated gene targeting. Cell (1993) 73(6):1155–64. doi: 10.1016/0092-8674(93)90644-6”.

Thus the correct sentence in the manuscript should be now

“The generation of J_H_^-/-^ and λ5^-/-^ mice on a C57BL/6 background has been previously reported (7-25)”

In the published article, the reference (7) in the results section at the sentence that reads “Developing B cells in these mice cannot undergo VDJH rearrangement and thus cannot form a μHC. They are devoid of preB, immature B, mature B, and plasma cells (7, 32)” was added incorrectly and should be deleted leaving only the correct reference “Gu H, Zou YR, Rajewsky K. Independent control of immunoglobulin switch recombination at individual switch regions evidenced through cre-loxP-mediated gene targeting. Cell (1993) 73(6):1155–64. doi: 10.1016/0092-8674(93)90644-6”. Thus the correct sentence in the manuscript should be now

“Developing B cells in these mice cannot undergo VDJ_H_ rearrangement and thus cannot form a μHC. They are devoid of preB, immature B, mature B, and plasma cells (25)”.

The authors apologize for this[/these] error[s] and state that this does not change the scientific conclusions of the article in any way. The original article has been updated.

