# Supplementary figures and images for: Loss of early B cell protein λ5 decreases bone mass and accelerates skeletal aging
Source: Front Immunol. 2022 Sep 14;13:906649. doi: 10.3389/fimmu.2022.906649 (PMC9516392; doi:10.3389/fimmu.2022.906649)

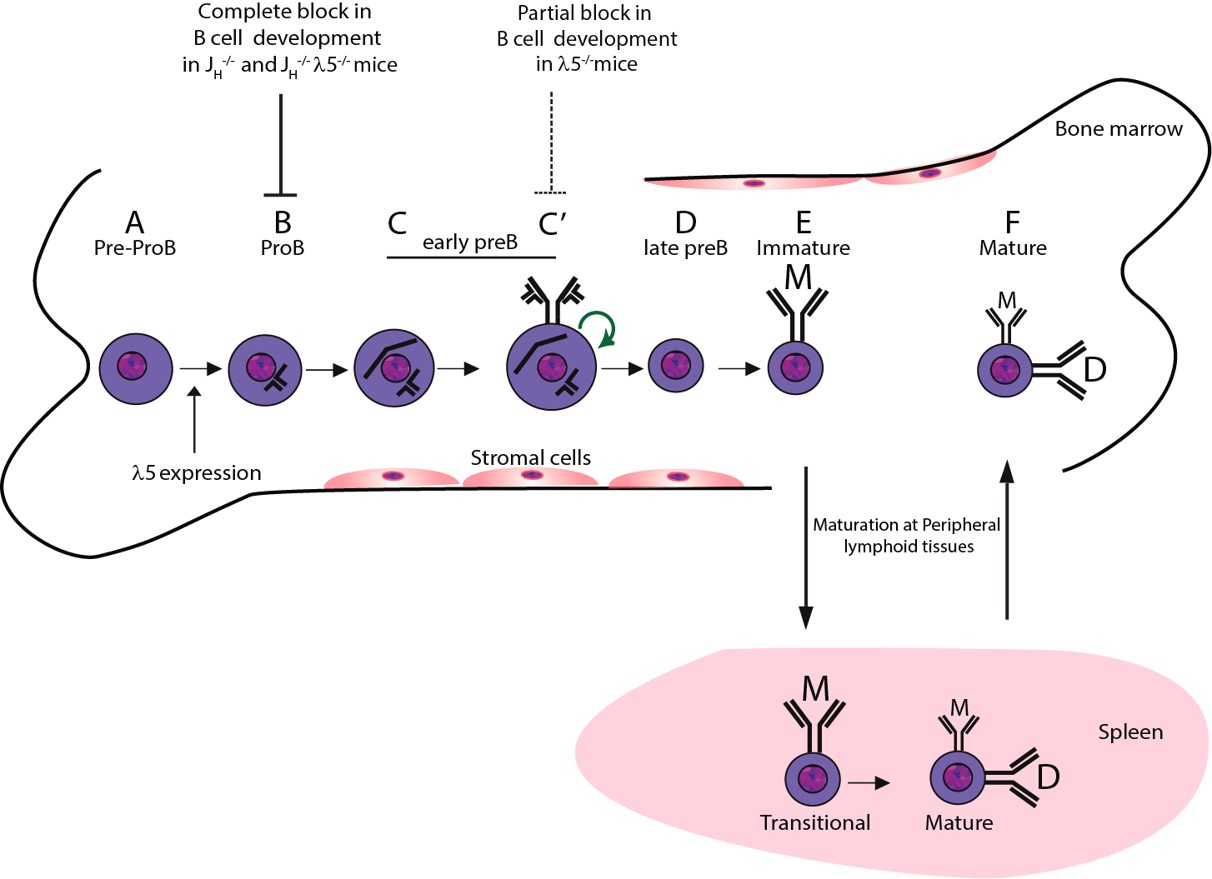

Supplement: Supplementary Figure 1 — Stages of B cell development in the bone marrow and periphery and impairment of B cell development in the mutant mice used in this study. B cells develop in the bone marrow and can be categorized as Hardy (48) fractions (A–E). B cells mature in the periphery (Transitional and Mature B cells). Expression of λ5 begins at Hardy fraction (B), µHC appears at (C) but cannot be formed in the JH -/- mice due to the failure of VDJH rearrangement. Surface preBCR expression, composed of µHC and SLC (λ5 plus VpreB), appears at C’. The stage and completeness of the blocks in B cell development in the absence of λ5 and/or JH are shown at the top of the figure. The dashed line represents a partial block in B cell development. [file Image_1.jpeg]

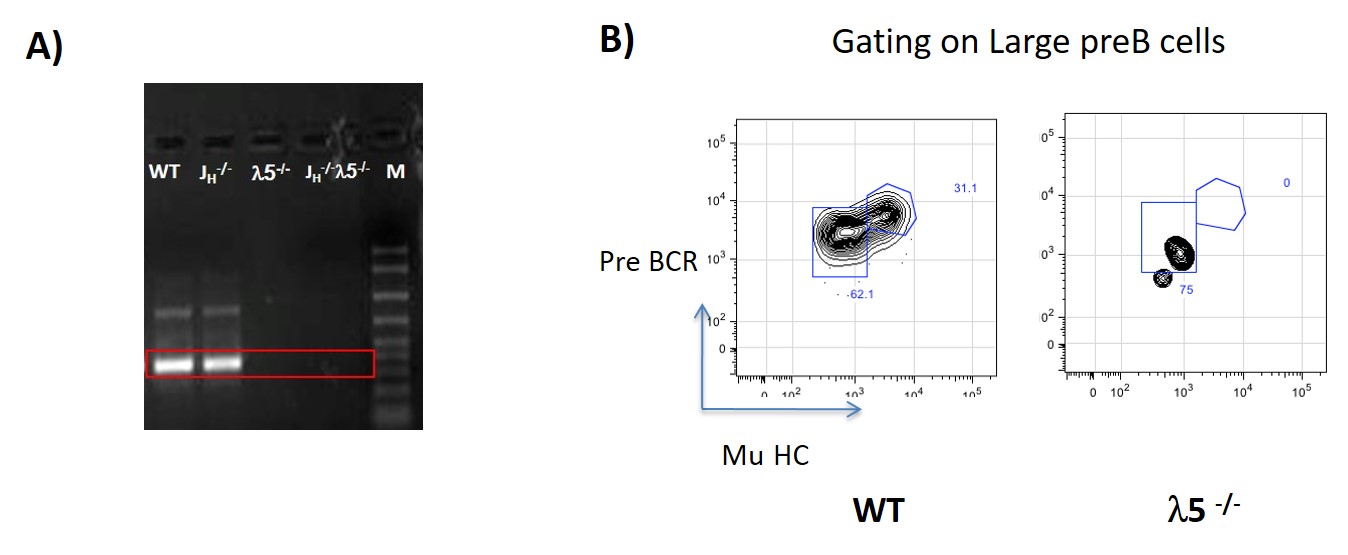

Supplement: Supplementary Figure 2 — Mouse genotyping and confirmation of the absence of preBCR expression in λ5-/- mice. (A) Genotyping. The λ5 gene (Igll1) was amplified by specific primers. The presence of Igll1 was confirmed in WT and JH -/- mice (lane1 and 2, red box) while its absence was confirmed in λ5-/- and JH -/- λ5-/- mice (lanes 3 and 4). (B) Flow Cytometry. Flow cytometry was used to confirm the absence of preBCR expression in the mutant λ5- deficient strains. Early B cell subsets including preB cells were identified based on expression of surface markers including: CD19, B220, AA4.1, and BP-1. Cells were fixed and permeabilized for intracellular staining. Antibodies specific for the preBCR (SL156 clone) and Mu HC were used to confirm the presence of the preBCR-expressing cells in WT mice (31.1% of cells) and their absence both on the cell surface and intracellularly in λ5-/- mice. [file Image_2.jpeg]

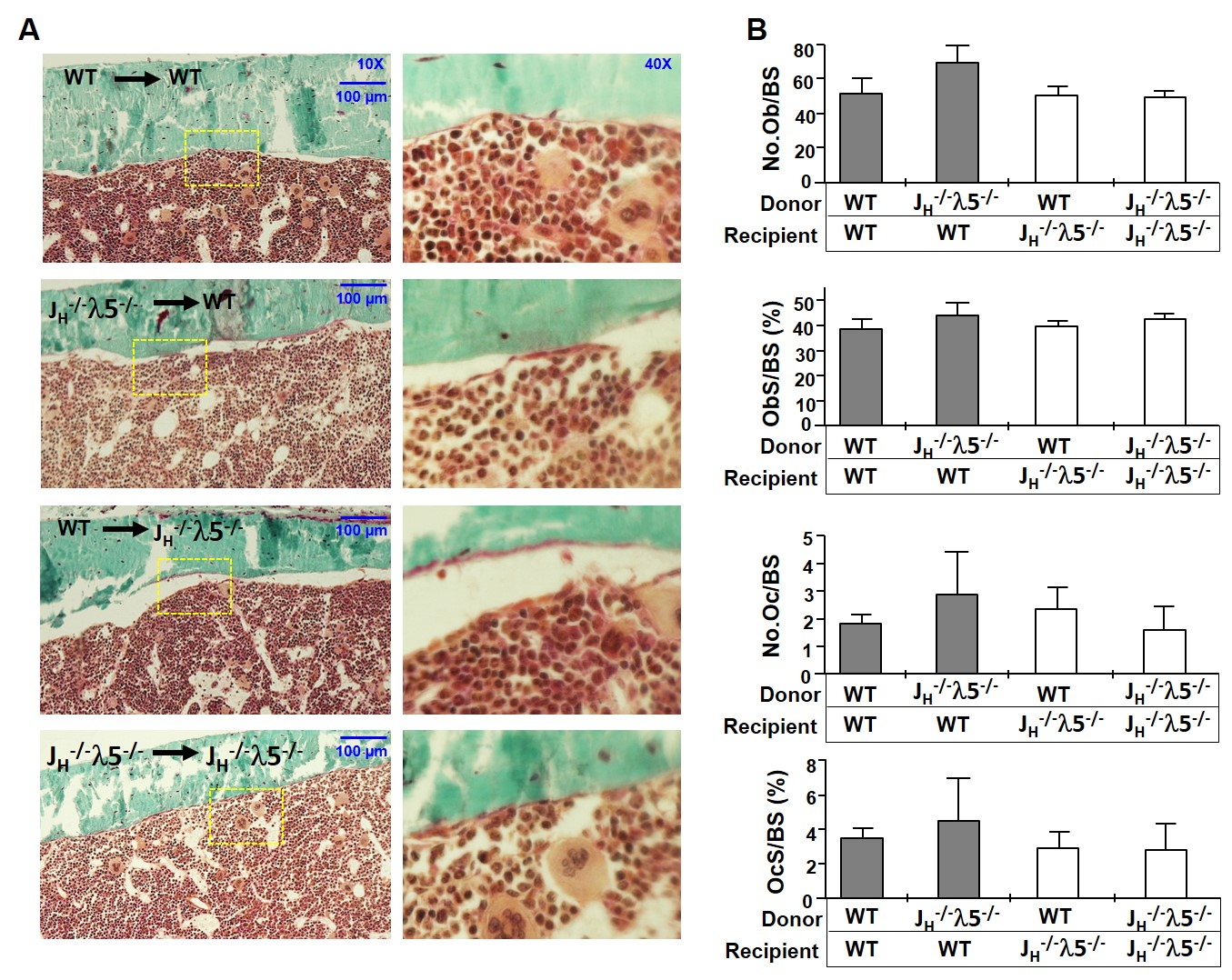

Supplement: Supplementary Figure 3 — Comparable numbers of osteoblasts and osteoclasts in bone marrow of recipient mice. (A) Undecalcified femurs from adoptive transfer mice were embedded in plastic, sectioned and stained with Masson’s trichrome. Representative images from the endosteal surface are shown at 10X magnification. The yellow boxed region is shown at 40X magnification. (B) Average histomorphometric values pooled from the indicated recipient mice (n=3-5 mice per group). No.Ob/BS=Number of osteoblasts per bone surface, ObS/BS=Osteoblast surface per bone surface, No.Oc/BS=number of osteoclasts per bone surface and OcS/Bs=Osteoclast surface per bone surface. Statistical significance was calculated by AOVA. Scale bar: 100µm. Data were obtained from n=3-5 mice per group. [file Image_3.jpg]
